# Association of metabolic dysfunction-associated fatty liver disease, type 2 diabetes mellitus, and metabolic goal achievement with risk of chronic kidney disease

**DOI:** 10.3389/fpubh.2022.1047794

**Published:** 2022-11-07

**Authors:** Weitao Su, Minhui Chen, Ling Xiao, Shanshan Du, Lihua Xue, Ruimei Feng, Weimin Ye

**Affiliations:** ^1^School of Public Health, Fujian Medical University, Fuzhou, China; ^2^Department of Ultrasonography, Fuqing Hospital, Fuqing, China; ^3^Department of Medical Epidemiology and Biostatistics, Karolinska Institutet, Stockholm, Sweden

**Keywords:** albuminuria, chronic kidney disease, estimated glomerular filtration rate, metabolic dysfunction-associated fatty liver disease, metabolic goal achievement, type 2 diabetes mellitus, urine albumin-to-creatinine ratio

## Abstract

**Background:**

Although type 2 diabetes mellitus (T2DM) plays a significant role in the association between metabolic dysfunction-associated fatty liver disease (MAFLD) and chronic kidney disease (CKD), how T2DM development and glycemic deterioration affect CKD and its renal function indicators, estimated glomerular filtration rate (eGFR) and urine albumin-to-creatinine ratio (UACR), remains unknown. We aimed to assess the association between MAFLD, along with T2DM, and risk of CKD, and then evaluate the effect of metabolic goal achievement in MAFLD on the risk of CKD.

**Methods:**

In this cross-sectional study, 5,594 participants were included. Multivariate logistic regression and linear regression were used to examine the association between MAFLD with its T2DM status and metabolic goal achievement and risk of CKD, as well as eGFR and UACR.

**Results:**

The MAFLD group had a higher prevalence of CKD (16.2 vs. 7.6%, *P* < 0.001) than the non-MAFLD group. MAFLD was independently associated with an increased risk of CKD (odds ratio [OR]: 1.35, 95% CI: 1.09–1.67) and increased eGFR and UACR. Among the three MAFLD subtypes, only the T2DM subtype exhibited significant associations with increased risk of CKD (OR: 2.85, 95% CI: 2.24–3.63), as well as increased eGFR and UACR. Glycemic deterioration in MAFLD was dose-dependently associated with an increased risk of CKD (*P*-trend < 0.001). Achieved metabolic goals in MAFLD decreased the risk of CKD, eGFR, and UACR; MAFLD with 2 or 3 achieved metabolic goals was not significantly associated with the risk of CKD (OR: 0.81, 95% CI: 0.59–1.12) and albuminuria.

**Conclusion:**

MAFLD was independently associated with an increased risk of CKD, as well as increased eGFR and UACR. This association is strongly driven by T2DM status. Glycemic deterioration in MAFLD was dose-dependently associated with an increased risk of CKD. Achieved metabolic goals in MAFLD decreased the risk of CKD by reducing the risk of albuminuria.

## Introduction

Nonalcoholic fatty liver disease (NAFLD) represents the most common chronic liver disorder worldwide, with a prevalence of 29.2% in China (1, 2). NAFLD is closely associated with metabolic disorders, such as type 2 diabetes mellitus (T2DM) (3), hypertension (4), and obesity (5), and other extra-hepatic diseases, such as chronic kidney disease (CKD) (6) and cardiovascular disease (CVD) (7). The newly propagated name, metabolic dysfunction-associated fatty liver disease (MAFLD), might be a more appropriate term to describe the liver disease associated with underlying metabolic dysfunction (8). The association between MAFLD and other diseases is still not fully understood.

Chronic kidney disease, another leading public health problem affecting nearly 10% of the Chinese population (9), is defined by estimated glomerular filtration rate (eGFR) and urine albumin-to-creatinine ratio (UACR) (10). Diabetes is the leading cause of CKD, and CKD is considered to share common metabolic risk factors with NAFLD, such as T2DM (6). The association between NAFLD and CKD is established in several epidemiological studies (11–15), two of which are based on patients with diabetes (11, 12). The risk of CKD progression increased with the severity of NAFLD in patients with T2DM (11). Putative mechanisms linking NAFLD with CKD include T2DM and metabolic syndrome, dysbiosis and perturbed intestinal function, and aging (6). Recent studies have shown that MAFLD is independently associated with CKD (16–18). Although T2DM plays a significant role in the association between NAFLD and CKD, how T2DM development and glycemic deterioration affect CKD and its renal function indicators, eGFR and UACR, remains unknown.

Metabolic dysfunction-associated fatty liver disease criteria identify an additional portion of people with more metabolic comorbidities and a higher risk of CKD compared to NAFLD (17, 19), so metabolic management is more important to reduce the risk of CKD than before. “ABCs” metabolic goal, including glycated hemoglobin A1c (HbA1c), blood pressure (BP), and low-density lipoprotein cholesterol (LDL-C) (20, 21), was first proposed to alleviate the complications of diabetes. A recent study revealed that metabolic goal achievement among NAFLD reduced the risk of CKD, and there was a significant interaction between poor glycemic control and NAFLD on the risk of CKD (12). However, whether overall and specific metabolic goal achievement among MAFLD has effects on CKD, as well as eGFR and UACR, still needs to be explored.

Therefore, we conducted a cross-sectional study based on the general population in southern China to evaluate the associations of MAFLD and its T2DM status with CKD, eGFR, and UACR, and then to check whether metabolic goal achievement among MAFLD reduces the risk of CKD.

## Materials and methods

### Study population

**Figure 1:**
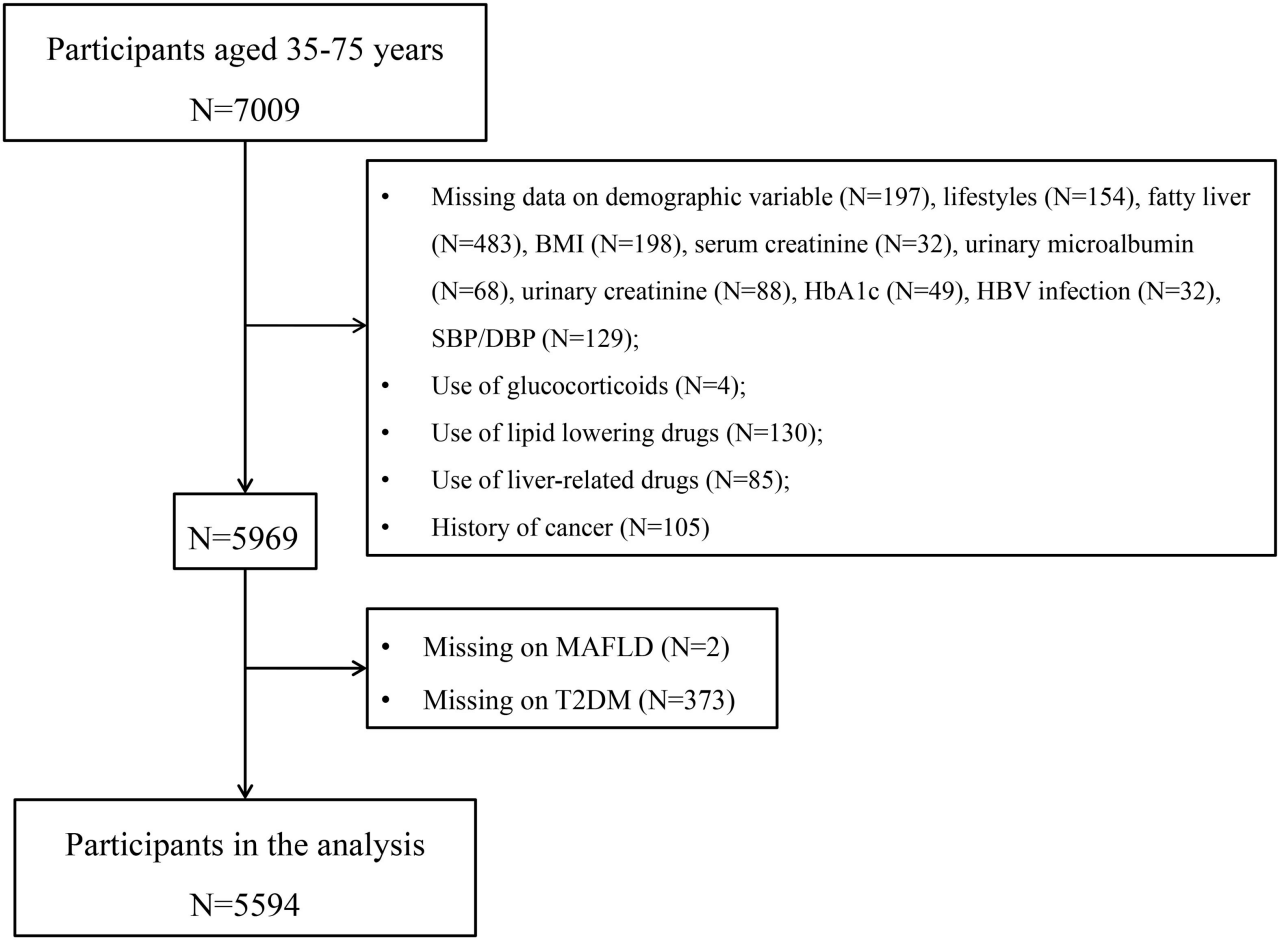
Flowchart of selection of study participants.

The study was based on baseline survey data of Fuqing Cohort from the general population of Fuqing City in southeast China. From July 2020 to June 2021, 7,009 eligible participants aged 35–75 years who had lived in Fuqing City for at least 5 years were recruited. After exclusion, a total of 5,594 participants were included for analysis (Figure 1). This study was conducted in compliance with the regulations of the Declaration of Helsinki and was approved by the Ethical Committee of Fujian Medical University (approval number [2020-58]). All participants provided written informed consent.

### Data collection

Face-to-face interviews for study participants were performed by a team of trained interviewers using an electronic structured questionnaire. The following information was collected: demographics, smoking, alcohol and tea drinking, physical activities, medical history, and medication use.

Height, weight, waist circumference, hip circumference, systolic blood pressure (SBP), and diastolic blood pressure (DBP) were measured by trained staff. Body mass index (BMI) was calculated as body weight in kilograms divided by height squared in meters (kg/m^2^). BP was measured using an electronic sphygmomanometer (OMRON U30; Omron Company, Kyoto, Japan) according to a standard protocol. The average of the three readings was calculated for analysis. Daily physical metabolic equivalent (MET/day) was calculated for the quantification of physical activity intensity.

Type 2 diabetes mellitus was defined as a fasting plasma glucose level ≥ 7.0 mmol/L or 2-h post-load glucose level ≥ 11.1 mmol/L or HbA1c ≥ 6.5% or use of antidiabetic drugs or self-reported history of diabetes. Prediabetes was defined as nondiabetic individuals having a fasting plasma glucose level of 5.6–6.9 mmol/L or 2-h post-load glucose level of 7.8–11.1 mmol/L or HbA1c of 5.7%−6.4%. Hypertension was defined as mean SBP ≥ 140 mmHg or mean DBP ≥ 90 mmHg or use of hypotension.

Overnight fasting venous blood samples and random urine samples were collected. Serum creatinine, glucose level, lipid profile, urinary creatinine, and microalbumin were measured using standard laboratory procedures (Toshiba automatic biochemical analyzer, TBA-120FR, Japan). HbA1c was measured using a high-performance liquid chromatography method (fully automated glycohemoglobin (HbA1c) analyzer, HA8180, Japan). Urinary creatinine and microalbumin were used to calculate UACR.

Abdominal ultrasonography was performed using an ultrasonic instrument (Prosoundα7, Hitachi Medical Company, Tokyo, Japan) by experienced ultrasonographists.

### Definition of metabolic dysfunction-associated fatty liver disease

Metabolic dysfunction-associated fatty liver disease was defined as evidence of fatty liver based on ultrasound with at least one of the following three criteria (8): (1) overweight or obesity (BMI ≥ 23.0 kg/m^2^ in Asians); (2) T2DM; and (3) metabolic dysregulation among non-overweight individuals (BMI < 23.0 kg/m^2^ in Asians). Metabolic dysregulation was defined as the presence of at least two of the following metabolic risk abnormalities: (1) waist circumference ≥ 90 cm for Asian men and 80 cm for Asian women; (2) BP ≥ 130/85 mmHg or specific drug treatment; (3) triglycerides (TG) ≥ 1.70 mmol/L or specific drug treatment; (4) high-density lipoprotein cholesterol (HDL-C) < 1.0 mmol/L for men and < 1.3 mmol/L for women; (5) prediabetes; (6) insulin resistance (HOMA-IR) score ≥ 2.5; and (7) plasma high-sensitivity C-reactive protein (hs-CRP) level > 2 mg/L.

### Definition of CKD

The Chronic Kidney Disease Epidemiology Collaboration (CKD-EPI) equation was used to calculate eGFR(22). The formula is given as follows: eGFR = 141 × min (Scr/κ, 1)^α^ × max (Scr/κ, 1)^−1.209^ × 0.993^Age^ × 1.018 [if female], where Scr is serum creatinine concentration in milligrams per deciliter, κ is 0.9 for males and 0.7 for females, α is −0.411 for males and −0.329 for females, min represents the minimum of 1 or Scr/κ, and max represents the maximum of 1 or Scr/κ. CKD was defined as eGFR < 60 ml/min/1.73 m^2^ or the presence of albuminuria. Albuminuria was defined as UACR ≥ 30 mg/g.

### Definition of ABCs' metabolic goal

“ABCs” metabolic goal was defined as HbA1c < 6.5% (A), SBP/DBP < 140/90 mmHg (B), and LDL-C < 100 mg/dl (C) (12, 20, 23).

### Statistical analysis

Continuous variables were expressed as median (25th and 75th percentiles) due to skewed distribution, and categorical variables were presented as frequency (percentage). Differences between non-MAFLD group and MAFLD group were assessed using the Wilcoxon rank sum test for continuous variables and Pearson's chi-squared test for categorical variables.

Multivariate logistic regression was used to estimate odds ratios (ORs) and corresponding 95% confidence intervals (95% CIs) for associations between MAFLD and CKD. Multivariate linear regression was used to estimate regression coefficients (βs) and corresponding 95% CIs for associations of MAFLD with eGFR and UACR. UACR values were log-transformed for analysis. The following models were constructed. Model 1 was unadjusted. Model 2 was adjusted for age, sex, and BMI. Model 3 was further adjusted for education level (0/1–6/7–9/≥10 years), occupation (farmer or unemployment/worker/sales or service/office job/others), alcohol drinking (never/former/current), tea drinking (never/former/current), HBV infection (yes/no), T2DM (yes/no, removed when conflicting with independent variable), and physical activity (MET/day).

Subgroup analysis was performed to assess potential effect modifiers by stratifying sex, age (< 65/≥65 years), BMI (< 28/≥28 kg/m^2^), alcohol drinking (never/ever), tea drinking (never/ever), smoking (never/ever), HBV infection, T2DM, hypertension, and dyslipidemia.

Statistical significance was defined as a 2-tailed *P* < 0.05. All statistical analyses were conducted using R version 4.0.5 (Foundation, Vienna, Austria).

## Results

### General characteristics of participants

The general characteristics of 5,594 participants are presented in Table 1. The median age of the participants was 57 (50, 65) years and 35.1% were men. Of them, 1,878 (33.6%) and 589 (10.5%) were diagnosed with MAFLD and CKD.

**Table 1:** General characteristics of subjects with and without MAFLD.

	**Total**	**Non-MAFLD**	**MAFLD**	**P value**
	***N* = 5,594**	***N* = 3,716**	***N* = 1,878**	
Age, median (25, 75th percentiles), year,	57 (50,65)	57 (49,65)	58 (52,66)	**<** **0.001**
Male, n (%)	1,961 (35.1)	1,275 (34.3)	686 (36.5)	0.101
Education level, years				0.114
0	1,921 (34.3)	1,245 (33.5)	676 (36.0)	
1-6	1,900 (34.0)	1,273 (34.3)	627 (33.4)	
7-9	1,303 (23.3)	894 (24.1)	409 (21.8)	
≥10	470 (8.4)	304 (8.2)	166 (8.8)	
Occupation				**0.028**
Farmer or unemployment	4,074 (72.8)	2,684 (72.2)	1,390 (74.0)	
Worker	590 (10.5)	425 (11.4)	165 (8.8)	
Sales or service	358 (6.4)	242 (6.5)	116 (6.2)	
Office job	511 (9.1)	326 (8.8)	185 (9.9)	
Other	61 (1.1)	39 (1.0)	22 (1.2)	
Alcohol drinking status				0.266
Never	4,976 (89.0)	3,320 (89.3)	1,656 (88.2)	
Former	186 (3.3)	114 (3.1)	72 (3.8)	
Current	432 (7.7)	282 (7.6)	150 (8.0)	
Smoking status				0.372
Never	4,129 (73.8)	2,762 (74.3)	1,367 (72.8)	
Former	491 (8.8)	314 (8.4)	177 (9.4)	
Current	974 (17.4)	640 (17.2)	334 (17.8)	
Tea drinking status				**<** **0.001**
Never	4,270 (76.3)	2,926 (78.7)	1,344 (71.6)	
Former	61 (1.1)	34 (0.9)	27 (1.4)	
Current	1,263 (22.6)	756 (20.3)	507 (27.0)	
T2DM, n (%)	1,248 (22.3)	515 (13.9)	733 (39.0)	**<** **0.001**
Hypertension, n (%)	2,609 (46.6)	1,447 (38.9)	1,162 (61.9)	**<** **0.001**
Hyperlipidemia, n (%)	1,986 (35.5)	1,118 (30.1)	868 (46.2)	**<** **0.001**
HBV infection, n (%)	787 (14.1)	590 (15.9)	197 (10.5)	**<** **0.001**
BMI, kg/m^2^	23.9 (21.8,26.0)	22.7 (21.0,24.6)	26.2 (24.6,28.2)	**<** **0.001**
Waist circumference, cm	82.8 (76.2,89.2)	79.4 (73.8,85.0)	89.6 (84.1,95.0)	**<** **0.001**
Hip circumference, cm	93.7 (89.8,98.0)	92 (88.4,95.8)	97.2 (93.6,101.2)	**<** **0.001**
SBP, mmHg	131.8 (119.0,146.5)	128.5 (116.5,143.0)	138.5 (125.5,152.9)	**<** **0.001**
DBP, mmHg	84.0 (77.0,92.0)	82.5 (75.5,89.5)	88.0 (81.0,95.5)	**<** **0.001**
Uric acid, umol/L	341.5 (291.5,404.5)	329.7 (282.9,389.4)	368.6 (314.9,433.6)	**<** **0.001**
Creatinine, umol/L	61.7 (54.5,72.4)	62.0 (55.2,71.8)	60.7 (53.1,73.7)	**0.015**
eGFR, mL/min/1.73 m^2^	97.17 (90.08,104.07)	97.18 (89.97,104.19)	97.15 (90.27,103.75)	0.4328
eGFR category				0.476
eGFR < 60	49 (0.9)	36 (1.0)	13 (0.7)	
60 ≤ eGFR < 90	1,333 (23.8)	894 (24.1)	439 (23.4)	
eGFR ≥90	4,212 (75.3)	2,786 (75.0)	1,426 (75.9)	
UACR, mg/g	7.22 (4.46,13.46)	6.57 (4.24,11.35)	9.09 (5.26,18.14)	**<** **0.001**
Albuminuria, n (%)	561 (10.0)	261 (7.0)	300 (16.0)	**<** **0.001**
CKD, n (%)	589 (10.5)	284 (7.6)	305 (16.2)	**<** **0.001**
FPG, mmol/L	4.95 (4.58,5.46)	4.84 (4.52,5.25)	5.24 (4.77,6.13)	**<** **0.001**
Postprandial GLU, mmol/L	7.25 (6.00,9.01)	6.92 (5.80,8.41)	8.23 (6.67,10.48)	**<** **0.001**
HbA1c, %	5.9 (5.7,6.2)	5.8 (5.6,6.0)	6.1 (5.8,6.6)	**<** **0.001**
TC, mmol/L	5.65 (4.96,6.36)	5.58 (4.90,6.27)	5.78 (5.11,6.51)	**<** **0.001**
TG, mmol/L	1.11 (0.82,1.56)	0.98 (0.74,1.29)	1.47 (1.09,2.09)	**<** **0.001**
HDL-C, mmol/L	1.57 (1.36,1.83)	1.64 (1.43,1.90)	1.45 (1.25,1.64)	**<** **0.001**
LDL-C, mmol/L	3.17 (2.70,3.69)	3.12 (2.66,3.60)	3.30 (2.82,3.79)	**<** **0.001**
MET/day	9.95 (6.65,16.05)	10 (6.65,16.60)	9.79 (6.65,15.09)	0.4014

All continuous variables were expressed as median (25, 75th percentiles), categorical variables were presented as frequency (percentage).

BMI, body mass index; CKD, chronic kidney disease; DBP, diastolic blood pressure; eGFR, estimated glomerular filtration rate; FPG, fasting plasma glucose; GLU, plasma glucose; HbA1c, glycated hemoglobin A1c; HBV, hepatitis B virus; HDL-C, high-density lipoprotein cholesterol; LDL-C, low-density lipoprotein cholesterol; MAFLD, metabolic associated fatty liver disease; MET/day, daily physical metabolic equivalent; SBP, systolic blood pressure; T2DM, type 2 diabetes mellitus; TC, total cholesterol; TG, triglyceride; UACR, urinary albumin to creatinine ratio. Boldface type indicates statistical significance (*P* value < 0.05).

Compared to the non-MAFLD group, participants with MAFLD were more likely to be older, men, to have significantly higher levels of BMI, waist circumference, hip circumference, SBP, DBP, UACR, fasting and postprandial plasma glucose, HbA1c, TC, TG, and LDL-C. In addition, the prevalence of T2DM, hypertension, hyperlipidemia, albuminuria, and CKD in the MAFLD group is significantly higher than that in the non-MAFLD group (Table 1).

### Association of MAFLD with CKD, EGFR, and UACR

As shown in Table 3, MAFLD was independently associated with an increased risk of CKD (OR: 1.35, 95% CI: 1.09–1.67) after adjusting for age, sex, BMI, education level, occupation, alcohol drinking, tea drinking, HBV infection, T2DM, and MET/day (Table 2). Considering the heterogeneity of MAFLD, MAFLD was categorized into three subtypes based on the definition (24, 25). A significant association was observed only in the T2DM subtype (OR: 2.85, 95% CI: 2.24–3.63). The same analysis was performed with eGFR and UACR, two indexes in the definition of CKD, as outcomes. Linear regression analysis showed that MAFLD was independently associated with increased eGFR (β: 1.37, 95% CI: 0.73–2.01) and UACR (β: 0.15, 95% CI: 0.09–0.21) (Supplementary Table S1). MAFLD subtype analysis showed that a significant association for eGFR was observed in the overweight/obesity subtype (β: 0.94, 95% CI: 0.19–1.69) and T2DM subtype (β: 3.10, 95% CI: 2.25–3.95), whereas for UACR, a significant association was observed only in the T2DM subtype (β: 0.53, 95% CI: 0.45–0.61). Collectively, these data suggest that T2DM is a crucial factor in the association between MAFLD and CKD.

**Table 2:** Association between MAFLD subgroups and CKD.

	**No**.	**CKD**	**Model 1^†^ (OR and 95% CI)**	**Model 2^‡^ (OR and 95% CI)**	**Model 3^§^(OR and 95% CI)**
Non-MAFLD	3,716	284	1.00 (ref)	1.00 (ref)	1.00 (ref)
MAFLD	1,878	305	**2.34 (1.97,2.78)**	**1.74 (1.42,2.13)**	**1.35 (1.09,1.67)**
T2DM (-)^¶^ & overweight/obesity (BMI>23)	1,046	106	**1.36 (1.08,1.72)**	1.08 (0.83,1.40)	1.07 (0.82,1.40)
T2DM (-)^¶^ & BMI < 23 & ≥2 metabolic disorders	99	9	1.21 (0.60,2.42)	1.26 (0.63,2.54)	1.22 (0.60,2.45)
T2DM (+)^††^	733	190	**4.23 (3.45,5.19)**	**2.89 (2.27,3.67)**	**2.85 (2.24,3.63)**

BMI, body mass index; CI, confidence interval; CKD, chronic kidney disease; MAFLD, metabolic associated fatty liver disease; OR, odds ratio; T2DM, type 2 diabetes mellitus.^†^Unadjusted model.^‡^Age, sex and BMI adjusted. ^§^Adjusted for age, sex, BMI, education level, occupation, alcohol drinking status, tea drinking status, HBV infection, T2DM (removed in MAFLD subgroup analysis) and MET/day. ^¶^Absence of T2DM.^††^Presence of T2DM. Boldface type indicates statistical significance (*P* value < 0.05).

**Table 3:** Association of MAFLD and T2DM with CKD.

	**No**.	**CKD**	**Model 1^†^ (OR and 95% CI)**	**Model 2^‡^** **(OR and 95% CI)**	**Model 3^§^(OR and 95% CI)**
Combination of MAFLD and T2DM		
MAFLD (-)^¶^ & T2DM (-)^††^	3,201	191	1.00 (ref)	1.00 (ref)	1.00 (ref)
MAFLD (+)^‡‡^ & T2DM (-)^††^	1,145	115	**1.76 (1.38,2.24)**	**1.41 (1.08,1.84)**	**1.40 (1.07,1.83)**
MAFLD (-)^¶^ & T2DM (+)^§§^	515	93	**3.47 (2.66,4.54)**	**2.87 (2.19,3.78)**	**2.87 (2.18,3.78)**
MAFLD (+)^‡‡^ & T2DM (+)^§§^	733	190	**5.51 (4.42,6.88)**	**3.76 (2.93,4.84)**	**3.72 (2.88,4.79)**
P-trend			<**0.001**	<**0.001**	<**0.001**
					
MAFLD subgroups by different T2DM status		
Non-MAFLD	3,716	284	1.00 (ref)	1.00 (ref)	1.00 (ref)
MAFLD without T2DM	1,145	115	**1.35 (1.07,1.69)**	1.10 (0.85,1.41)	1.09 (0.84,1.40)
MAFLD with normal glucose	135	9	0.86 (0.43,1.72)	0.86 (0.42,1.72)	0.84 (0.42,1.70)
MAFLD with prediabetes	1,010	106	**1.42 (1.12,1.79)**	1.12 (0.86,1.45)	1.11 (0.85,1.44)
MAFLD with T2DM	733	190	**4.23 (3.45,5.19)**	**2.87 (2.26,3.63)**	**2.83 (2.23,3.58)**
MAFLD with newly diagnosed T2DM^¶¶^	420	89	**3.25 (2.50,4.23)**	**2.23 (1.67,2.98)**	**2.21 (1.65,2.96)**
MAFLD with pre-existing T2DM^†††^	313	101	**5.76 (4.41,7.51)**	**3.82 (2.86,5.11)**	**3.75 (2.80,5.02)**
P-trend			<**0.001**	<**0.001**	**<** **0.001**

CI, confidence interval; CKD, chronic kidney disease; MAFLD, metabolic associated fatty liver disease; OR, odds ratio; T2DM, type 2 diabetes mellitus.^†^Unadjusted model.^‡^Age, sex, and BMI adjusted. ^§^Adjusted for age, sex, BMI, education level, occupation, alcohol drinking status, tea drinking status, HBV infection, and MET/day. ^¶^Absence of MAFLD.^†^^†^Absence of T2DM.^‡‡^Presence of MAFLD. ^§§^Presence of T2DM. ^¶¶^Newly diagnosed T2DM is defined by laboratory indicators without pre-existing T2DM, meaning relative short duration of T2DM.^†††^Pre-existing T2DM is defined as self-reported T2DM and use of antidiabetic drug, meaning relative long duration of T2DM. Boldface type indicates statistical significance (*P* value < 0.05).

Subgroup analysis found no significant interaction in the association between MAFLD and CKD (Supplementary Table S2). Significant interactions were observed for eGFR and UACR as outcomes (Supplementary Table S3). For eGFR, the association was stronger for individuals who were female, nonsmokers, and had T2DM. For UACR, the association was stronger for individuals who had T2DM and hypertension.

### Association of MAFLD and T2DM with CKD, EGFR, and UACR

To further study the role of T2DM in the association between MAFLD and CKD, MAFLD and T2DM were combined for analysis (Table 3). Using non-MAFLD without T2DM group as the reference, MAFLD without T2DM (OR: 1.40, 95% CI: 1.07–1.83), non-MAFLD with T2DM (OR: 2.87, 95% CI: 2.18–3.78), and MAFLD with T2DM (OR: 3.72, 95% CI: 2.88, 4.79) groups were all associated with an increased risk of CKD. To better understand the association between glycemic status in MAFLD and CKD, MAFLD was categorized by T2DM status. The unadjusted ORs of normal glucose group, prediabetes group, and T2DM group among MAFLD for CKD were 0.86 (95% CI: 0.43–1.72), 1.42 (95% CI: 1.12–1.79), and 4.23 (95% CI: 3.45–5.19), respectively. Only the T2DM group remained statistically significant after full adjustment (OR: 2.83, 95% CI: 2.23–3.58). Similarly, for eGFR and UACR, a significant association was observed in the T2DM group (Supplementary Table S4). The T2DM group was further divided into newly diagnosed T2DM and pre-existing T2DM to explore the effect of T2DM duration on CKD. The pre-existing T2DM group had a higher OR (OR: 3.75, 95% CI: 2.80–5.02) than newly diagnosed T2DM (OR: 2.21, 95% CI: 1.65–2.96), indicating that the risk of CKD increases with T2DM duration (Table 3).

### Association of MAFLD and metabolic goal achievement with CKD, EGFR, and UACR

**Figure 2:**
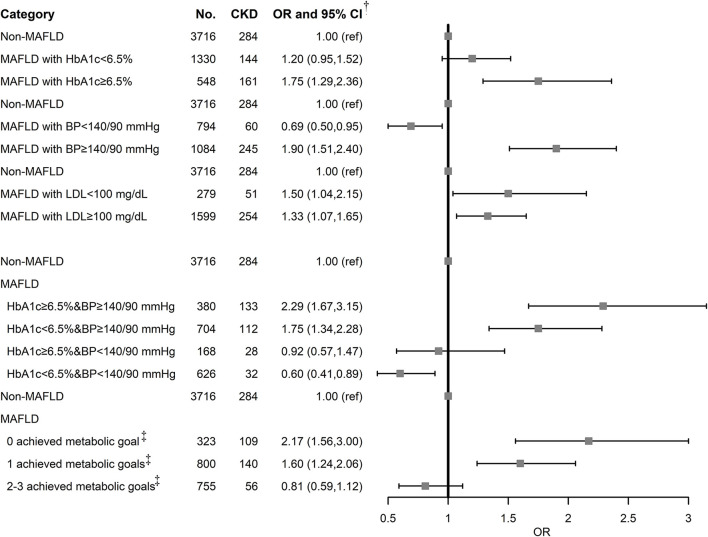
Association of MAFLD and metabolic goal achievement with CKD. BP, blood pressure; CI, confidence interval; CKD, chronic kidney disease; MAFLD, metabolic associated fatty liver disease; OR, odds ratio; T2DM, type 2 diabetes mellitus.^†^Adjusted for age, sex, BMI, education level, occupation, alcohol drinking status, tea drinking status, HBV infection, T2DM, and MET/day.^‡^Achieved metabolic goal is defined as HbA1c < 6.5%, SBP/DBP < 140/90 mmHg, and LDL-C < 100 mg/dl.

“ABCs” metabolic goal was introduced to study whether the metabolic goal achievement reduces the risk of CKD among MAFLD (Figure 2). MAFLD with poorly controlled HbA1c, BP, and LDL was associated with an increased risk of CKD. MAFLD with HbA1c (OR: 1.20, 95% CI: 0.95–1.52) and BP goal achievement (OR: 0.69, 95% CI: 0.50–0.95) decreased the risk of CKD, while LDL goal achievement has no effect on CKD (OR: 1.50, 95% CI: 1.04–2.15). Notably, BP goal achievement reversed the increased risk to a decreased one (OR: 0.69, 95% CI: 0.50–0.95), suggesting the importance of BP control to reduce CKD risk in subjects with MAFLD. A combination of HbA1c and BP control in MAFLD exhibited gradually decreased risks of CKD. The group achieving both HbA1c and BP control had the lowest risk (OR: 0.60, 95% CI: 0.41–0.89). With more achieved metabolic goals in MAFLD, the risk of CKD decreased more obviously. MAFLD with 2 or 3 achieved metabolic goals exhibited no statistically significant association with CKD (OR: 0.81, 95% CI: 0.59–1.12).

Using eGFR and UACR as outcomes, metabolic goal achievement in MAFLD significantly decreased eGFR and UACR (Supplementary Table S5). We also assessed the association with albuminuria, and the ORs were similar to those for CKD (Supplementary Table S6).

## Discussion

In this cross-sectional study based on the general population in southern China, we demonstrated that the presence of MAFLD significantly increased the risk of CKD by increasing both eGFR and UACR. This significant association was observed only in the T2DM subtype among MAFLD. Categorized by glycemic status, MAFLD with T2DM group rather than prediabetes and normal glucose groups was associated with an increased risk of CKD. Furthermore, metabolic goal achievement in MAFLD decreased the risk of CKD by reducing the risk of albuminuria.

In our population, the prevalence of MAFLD was 33.6%, higher than 29.2% and 26.1% in two studies based on the populations of southern China (26, 27). This may be attributed to demographic differences and increasing prevalence over time. The prevalence of eGFR < 60 ml/min/1.73 m^2^, albuminuria, and CKD was 0.9, 10.0, and 10.5%, respectively, compared to those of 1.7, 9.4, and 10.8% from a nationally representative sample of the Chinese adults (9).

Numerous studies have reported a significant association between NAFLD and CKD (6, 11–14). Recently, a significant association between MAFLD and CKD was established in cross-sectional and cohort studies (16–18). Our study further examined this association. To the best of our knowledge, MAFLD has great heterogeneity due to its definition and can be classified into different subtypes (24, 25). There is currently no study that explores the association between MAFLD subtypes and CKD. In our study, a significant association was observed only in the T2DM subtype among the three MAFLD subtypes. Furthermore, results indicated that the T2DM subtype significantly increased eGFR and UACR, two renal function indexes defining CKD. We speculated from the definition of CKD that increased albuminuria by the T2DM subtype in MAFLD exceeds increased eGFR, leading to CKD.

Diabetes is the leading cause of CKD, referred to as diabetic kidney disease (DKD), developing in around 40% of patients with T2DM (28). Recent evidence showed that MAFLD complicated with T2DM had a higher prevalence of albuminuria and CKD than MAFLD without T2DM (17). We observed a synergy effect of MAFLD and T2DM on CKD, as well as eGFR and UACR. Further categorized into normal glucose, prediabetes, and T2DM, only MAFLD with T2DM group was significantly associated with CKD. In addition, the pre-existing T2DM group had a higher risk of CKD than the newly diagnosed T2DM, suggesting that T2DM progression among MAFLD elevated the risk of CKD. MAFLD with glycemic deterioration was associated with increased eGFR and UACR. It is noteworthy that MAFLD with prediabetes was still associated with increased eGFR, rather than UACR. The natural pathway of DKD includes glomerular hyperfiltration, progressive albuminuria, declining GFR, and finally, end-stage renal disease (ESRD) (29). Glomerular hyperfiltration, characterized by increased GFR, was observed in prediabetes, early stage of DKD (29, 30), and NAFLD(31, 32), consistent with our findings that MAFLD with prediabetes and T2DM significantly increased eGFR. Research showed that baseline high eGFR or glomerular hyperfiltration was associated with faster decline over time and worse renal outcomes (30, 33, 34). Whether increased eGFR in MAFLD and T2DM will lead to faster decline and CKD development and progression needs to be explored in our follow-up studies. Albuminuria is present in around 30% of patients with T2DM and does not occur in the absence of hyperglycemia (29). MAFLD itself is also independently associated with an increased risk of albuminuria (17). Consistent with these studies, our data showed that only MAFLD with T2DM group significantly increased UACR, and UACR continued to increase with the duration of diabetes. Taken together, it is reasonable that we found that both eGFR and UACR were increased in the MAFLD with T2DM group.

As the new definition of MAFLD incorporates more metabolic risk factors and confers a high risk of CKD than NAFLD (17, 19), metabolic management is essential for reducing the risk of CKD. Our results demonstrated that the achievement of any 2 “ABCs” metabolic goals comprising HbA1c, BP, and LDL decreased the adverse effect of MAFLD on albuminuria and CKD risk to null. As we know, it is very difficult to achieve comprehensive metabolic control in practice, so it makes more sense to identify a specific metabolic goal that reduces enormous risks for diseases. Our findings revealed that MAFLD with glycemic and BP control, rather than LDL control, exhibited no statistically significant associations with CKD. According to the Global Burden of Disease study data from 1990 to 2016, diabetes and hypertension are the first two important causes of CKD(35). A meta-analysis of four randomized controlled trials (RCTs) showed that more intensive glucose control in adults with T2DM over 5 years reduced kidney events (36). Several long-term follow-up studies found that glycemic control in patients with diabetes reduced the risk of DKD and albuminuria (37, 38), in line with our findings. Research suggested that intensive BP control decreased the risk of incident CKD in patients with hypertension or diabetes (39). Interestingly, our study showed that an increased risk of CKD was reversed to a decreased one after BP control, suggesting BP serves as an important bridge between MAFLD and CKD. MAFLD with both well-controlled HbA1c and BP conferred the lowest risk compared to other groups. Similarly, there is evidence that a combination of poorly controlled blood glucose and BP was associated with a higher risk of incident CKD than having either alone (40, 41). Metabolic goal achievement in MAFLD significantly decreased eGFR and risk of albuminuria, suggesting that this decreased risk of CKD by metabolic goal achievement was basically attributed to decreased albuminuria. The real effect of metabolic goal achievement on eGFR may need to be assessed in our follow-up studies. Moreover, we found that MAFLD with HbA1c < 6.5% significantly reduced eGFR, consistent with studies showing that improved HbA1c mitigates glomerular hyperfiltration in the early-stage DKD (30).

The strength of our study is that we explored MAFLD with different glycemic statuses on the risk of CKD and identified the T2DM subtype which conferred the highest risk of CKD among MAFLD subtypes. Furthermore, the introduction of “ABCs” metabolic goal highlighted the importance of metabolic management in reducing the risk of CKD in MAFLD. We also used eGFR and UACR as outcomes to evaluate their impacts on CKD. However, there are limitations in this study. First, this cross-sectional study prevents us from making inferences about a causal relationship. Second, ultrasound, rather than a gold-standard liver biopsy, was performed to diagnose fatty liver in our study. Although insufficient accuracy is a problem, ultrasound is still the first choice in large-scale epidemiological studies for its accessibility and feasibility. Third, GFR and albuminuria have a dynamic, fluctuating progressive process (29), so only one sampling may not represent the real condition. Future follow-up studies are needed to explore the long-term changes in these renal function indexes and CKD development and progression among the three MAFLD subtypes. Whether increased eGFR in the T2DM subtype will lead to a faster decline and CKD progression is an interesting topic.

In conclusion, we demonstrated that MAFLD significantly increased the risk of CKD by increasing both eGFR and UACR. The T2DM subtype is the only MAFLD subtype driving this relationship. MAFLD with T2DM group rather than prediabetes and normal glucose groups was associated with an increased risk of CKD, as well as increased eGFR and UACR. Moreover, metabolic goal achievement in MAFLD significantly decreased the risk of CKD by reducing the risk of albuminuria.

## Data availability statement

The original contributions presented in the study are included in the article/Supplementary materials, further inquiries can be directed to the corresponding author.

## Ethics statement

The studies involving human participants were reviewed and approved by Fujian Medical University. The patients/participants provided their written informed consent to participate in this study.

## Author contributions

WS: conceptualization, data curation, formal analysis, methodology, and writing—original draft. LXi: conceptualization, investigation, validation, and writing—original draft. MC: formal analysis, visualization, and writing—review and editing. SD: data curation and project administration. LXu: investigation and validation. RF: data curation and writing—review and editing. WY: conceptualization, funding acquisition, project administration, and writing—review and editing. All authors contributed to the article and approved the submitted version.

## Funding

This study was jointly supported by the Government of Fuqing City (Grant No. 2019B003), the Department of Science and Technology of Fujian, China (Grant Nos. 2019Y9021 and 2019L3006), the National Natural Science Foundation of China (82103923), the Special Funds of Fujian Provincial Finance Department (Grant No. 2020czbz01), and the High-level Talents Research Start-Up Project of Fujian Medical University (Grant Nos. XRCZX2017035 and XRCZX2020034).

## Conflict of interest

The authors declare that the research was conducted in the absence of any commercial or financial relationships that could be construed as a potential conflict of interest.

## Publisher's note

All claims expressed in this article are solely those of the authors and do not necessarily represent those of their affiliated organizations, or those of the publisher, the editors and the reviewers. Any product that may be evaluated in this article, or claim that may be made by its manufacturer, is not guaranteed or endorsed by the publisher.
