# Supplementary material for: Association of metabolic dysfunction-associated fatty liver disease, type 2 diabetes mellitus, and metabolic goal achievement with risk of chronic kidney disease
Source: Front Public Health. 2022 Nov 7;10:1047794. doi: 10.3389/fpubh.2022.1047794 (PMC9676964; doi:10.3389/fpubh.2022.1047794)
Supplement: Supplementary file 1 [file Table_1.docx]

Supplementary Material

Supplementary table 1. Association of MAFLD subgroups with eGFR and UACR

|  | No. | eGFR | |  | UACR | |
| --- | --- | --- | --- | --- | --- | --- |
|  |  | Model 2^†^  (β and 95% CI) | Model 3^‡^  (β and 95% CI) |  | Model 2^†^  (β and 95% CI) | Model 3^‡^  (β and 95% CI) |
| Non-MAFLD | 3716 | Ref | Ref |  | Ref | Ref |
| MAFLD | 1878 | **1.61 (0.99,2.24)** | **1.37 (0.73,2.01)** |  | **0.24 (0.18,0.30)** | **0.15 (0.09,0.21)** |
| T2DM (-)^§^ & overweight/obesity (BMI>23) | 1046 | **0.88 (0.13,1.63)** | **0.94 (0.19,1.69)** |  | 0.06 (-0.01,0.13) | 0.05 (-0.02,0.12) |
| T2DM (-)^§^ & BMI<23 & ≥2 metabolic disorders | 99 | 0.44 (-1.46,2.35) | 0.48 (-1.42,2.39) |  | 0.10 (-0.08,0.28) | 0.09 (-0.09,0.27) |
| T2DM (+)^¶^ | 733 | **3.00 (2.16,3.85)** | **3.10 (2.25,3.95)** |  | **0.53 (0.45,0.61)** | **0.53 (0.45,0.61)** |

Abbreviations: BMI, body mass index; CI, confidence interval; eGFR, estimated glomerular filtration rate; MAFLD, metabolic associated fatty liver disease; T2DM, type 2 diabetes mellitus; UACR, urinary albumin to creatinine ratio. ^†^Age, sex and BMI adjusted. ^‡^Adjusted for age, sex, BMI, education level, occupation, alcohol drinking status, tea drinking status, HBV infection, T2DM (removed in MAFLD subgroup analysis) and MET/day. ^§^Absence of T2DM. ^¶^Presence of T2DM. Boldface type indicates statistical significance (P value < 0.05).

Supplementary table 2. Subgroup analysis on association between MAFLD and CKD

|  |  | No. | | Model 1^†^  (OR and 95% CI) | Model 2^‡^  (OR and 95% CI) | Model 3^§^  (OR and 95% CI) | P interaction |
| --- | --- | --- | --- | --- | --- | --- | --- |
| All |  | | 5594 | 2.34 (1.97,2.78) | 1.74 (1.42,2.13) | 1.35 (1.09,1.67) |  |
| Sex | Male | | 1961 | 2.12 (1.59,2.84) | 2.00 (1.40,2.84) | 1.55 (1.07,2.24) | 0.426 |
|  | Female | | 3633 | 2.47 (2.00,3.06) | 1.65 (1.28,2.12) | 1.31 (1.00,1.70) |  |
| Age | <65 | | 4067 | 2.37 (1.90,2.96) | 1.59 (1.22,2.07) | 1.26 (0.96,1.65) | 0.655 |
|  | ≥65 | | 1527 | 2.17 (1.65,2.87) | 2.02 (1.46,2.79) | 1.57 (1.11,2.22) |  |
| BMI | <28 | | 4942 | 2.12 (1.75,2.58) | 1.74 (1.39,2.17) | 1.32 (1.05,1.67) | 0.812 |
|  | ≥28 | | 652 | 1.91 (1.10,3.30) | 1.83 (1.05,3.19) | 1.46 (0.82,2.61) |  |
| Alcohol drinking | Never | | 4976 | 2.29 (1.91,2.75) | 1.66 (1.34,2.06) | 1.28 (1.02,1.60) | 0.176 |
|  | Ever | | 618 | 2.90 (1.68,5.02) | 2.70 (1.40,5.20) | 2.23 (1.14,4.35) |  |
| Tea drinking | Never | | 4270 | 2.26 (1.85,2.75) | 1.64 (1.29,2.07) | 1.27 (0.99,1.62) | 0.203 |
|  | Ever | | 1324 | 2.67 (1.87,3.82) | 2.03 (1.33,3.08) | 1.65 (1.07,2.54) |  |
| Smoking | Never | | 4129 | 2.43 (1.99,2.97) | 1.64 (1.30,2.08) | 1.30 (1.02,1.66) | 0.655 |
|  | Ever | | 1465 | 2.11 (1.51,2.96) | 2.15 (1.43,3.24) | 1.65 (1.08,2.54) |  |
| HBV | No | | 4807 | 2.40 (2.00,2.89) | 1.84 (1.48,2.29) | 1.44 (1.15,1.81) | 0.201 |
|  | Yes | | 807 | 1.84 (1.10,3.07) | 1.11 (0.60,2.04) | 0.81 (0.42,1.58) |  |
| T2DM | No | | 4346 | 1.76 (1.38,2.24) | 1.43 (1.08,1.91) | 1.41 (1.05,1.88) | 0.680 |
|  | Yes | | 1248 | 1.59 (1.20,2.10) | 1.33 (0.97,1.81) | 1.31 (0.96,1.80) |  |
| Hypertension | No | | 2985 | 1.50 (1.02,2.21) | 1.25 (0.79,1.97) | 1.02 (0.63,1.63) | 0.375 |
|  | Yes | | 2609 | 1.86 (1.52,2.28) | 1.63 (1.29,2.05) | 1.30 (1.02,1.65) |  |
| Dyslipidemia | No | | 3608 | 2.16 (1.69,2.75) | 1.50 (1.12,1.99) | 1.18 (0.88,1.59) | 0.713 |
|  | Yes | | 1986 | 2.14 (1.66,2.75) | 1.78 (1.33,2.39) | 1.40 (1.03,1.90) |  |

Abbreviations: BMI, body mass index; CI, confidence interval; CKD, chronic kidney disease; HBV, hepatitis B virus; MAFLD, metabolic associated fatty liver disease; OR, odds ratio; T2DM, type 2 diabetes mellitus. ^†^Unadjusted model. ^‡^Age, sex and BMI adjusted. ^§^Adjusted for age, sex, BMI, education level, occupation, alcohol drinking status, tea drinking status, HBV infection, T2DM and MET/day.

Supplementary table 3. Subgroup analysis on association of MAFLD with eGFR and UACR

|  |  |  | eGFR | | |  |  | UACR | |
| --- | --- | --- | --- | --- | --- | --- | --- | --- | --- |
|  |  | No. | | Model3 ^†^  (β and 95% CI) | P interaction |  |  | Model3 ^†^  (β and 95% CI) | P interaction |
| All |  | 5594 | | 1.37 (0.73,2.01) |  |  |  | 0.15 (0.09,0.21) |  |
| Sex | Male | 1961 | | 0.24 (-0.92,1.40) | **<0.001** |  |  | 0.18 (0.08,0.29) | 0.052 |
|  | Female | 3633 | | 2.30 (1.53,3.07) |  |  |  | 0.13 (0.05,0.20) |  |
| Age | <65 | 4067 | | 1.68 (0.92,2.44) | 0.076 |  |  | 0.12 (0.05,0.19) | 0.080 |
|  | ≥65 | 1527 | | 0.91 (-0.30,2.13) |  |  |  | 0.22 (0.10,0.34) |  |
| BMI | <28 | 4942 | | 1.36 (0.68,2.04) | 0.941 |  |  | 0.16 (0.10,0.22) | 0.727 |
|  | ≥28 | 652 | | 1.69 (-0.37,3.76) |  |  |  | 0.08 (-0.12,0.29) |  |
| Alcohol drinking | Never | 4976 | | 1.42 (0.74,2.11) | 0.102 |  |  | 0.13 (0.07,0.20) | 0.192 |
|  | Ever | 618 | | 0.97 (-0.90,2.84) |  |  |  | 0.23 (0.05,0.41) |  |
| Tea drinking | Never | 4270 | | 1.47 (0.73,2.21) | 0.457 |  |  | 0.14 (0.07,0.21) | 0.245 |
|  | Ever | 1324 | | 1.16 (-0.16,2.48) |  |  |  | 0.16 (0.04,0.28) |  |
| Smoking | Never | 4129 | | 1.93 (1.20,2.66) | **<0.001** |  |  | 0.13 (0.06,0.20) | 0.382 |
|  | Ever | 1465 | | 0.25 (-1.09,1.60) |  |  |  | 0.20 (0.08,0.32) |  |
| HBV | No | 4807 | | 1.16 (0.47,1.85) | 0.090 |  |  | 0.16 (0.09,0.22) | 0.583 |
|  | Yes | 807 | | 2.93 (1.16,4.69) |  |  |  | 0.08 (-0.10,0.25) |  |
| T2DM | No | 4346 | | 0.62 (-0.10,1.34) | **0.040** |  |  | 0.13 (0.07,0.20) | **0.030** |
|  | Yes | 1248 | | 2.95 (1.54,4.35) |  |  |  | 0.21 (0.06,0.36) |  |
| Hypertension | No | 2985 | | 0.84 (-0.04,1.72) | 0.797 |  |  | 0.04 (-0.03,0.11) | **<0.001** |
|  | Yes | 2609 | | 1.88 (0.93,2.83) |  |  |  | 0.16 (0.07,0.26) |  |
| Dyslipidemia | No | 3608 | | 1.23 (0.43,2.02) | 0.306 |  |  | 0.09 (0.02,0.17) | 0.070 |
|  | Yes | 1986 | | 2.00 (0.89,3.10) |  |  |  | 0.18 (0.08,0.29) |  |

Abbreviations: BMI, body mass index; CI, confidence interval; eGFR, estimated glomerular filtration rate; HBV, hepatitis B virus; MAFLD, metabolic associated fatty liver disease; T2DM, type 2 diabetes mellitus; UACR, urinary albumin to creatinine ratio. ^†^Adjusted for age, sex, BMI, education level, occupation, alcohol drinking status, tea drinking status, HBV infection, T2DM and MET/day. Boldface type indicates statistical significance (P value < 0.05).

Supplementary table 4. Association of MAFLD and T2DM with eGFR and UACR

|  | No. | eGFR | |  | UACR | |
| --- | --- | --- | --- | --- | --- | --- |
|  |  | Model 2^†^  (β and 95% CI) | Model 3^‡^  (β and 95% CI) |  | Model 2^†^  (β and 95% CI) | Model 3^‡^  (β and 95% CI) |
| Combination of MAFLD and T2DM |  |  |  |  |  |  |
| MAFLD (-)^§^ & T2DM (-)^¶^ | 3201 | Ref | Ref |  | Ref | Ref |
| MAFLD (+)^††^ & T2DM (-)^¶^ | 1145 | **0.95 (0.23,1.67)** | **1.02 (0.29,1.74)** |  | **0.12 (0.05,0.18)** | **0.11 (0.04,0.18)** |
| MAFLD (-)^§^ & T2DM (+)^‡‡^ | 515 | 0.80 (-0.10,1.70) | 0.83 (-0.07,1.73) |  | **0.35 (0.27,0.44)** | **0.35 (0.27,0.44)** |
| MAFLD (+)^††^ & T2DM (+)^‡‡^ | 733 | **3.06 (2.20,3.91)** | **3.16 (2.31,4.02)** |  | **0.60 (0.52,0.68)** | **0.59 (0.51,0.68)** |
| P-trend |  | **< 0.001** | **< 0.001** |  | **< 0.001** | **< 0.001** |
| MAFLD subgroups by different T2DM status | |  |  |  |  |  |
| Non-MAFLD | 3716 | Ref | Ref |  | Ref | Ref |
| MAFLD without T2DM | 1145 | **0.83 (0.12,1.54)** | **0.89 (0.17,1.60)** |  | 0.06 (-0.01,0.13) | 0.06 (-0.01,0.12) |
| MAFLD with normal glucose | 135 | -0.36 (-2.03,1.32) | -0.31 (-1.99,1.37) |  | -0.05 (-0.21,0.1) | -0.06 (-0.22,0.1) |
| MAFLD with prediabetes | 1010 | **0.98 (0.24,1.72)** | **1.04 (0.29,1.78)** |  | **0.07 (0.00,0.14)** | **0.07 (0.00,0.14)** |
| MAFLD with T2DM | 733 | **2.91 (2.08,3.75)** | **3.01 (2.17,3.85)** |  | **0.54 (0.46,0.61)** | **0.53 (0.45,0.61)** |
| MAFLD with newly diagnosed T2DM^§§^ | 420 | **2.32 (1.29,3.35)** | **2.40 (1.37,3.43)** |  | **0.37 (0.27,0.46)** | **0.36 (0.26,0.46)** |
| MAFLD with pre-existing T2DM^¶¶^ | 313 | **3.73 (2.57,4.88)** | **3.85 (2.69,5.01)** |  | **0.76 (0.65,0.87)** | **0.76 (0.65,0.87)** |
| P‐trend |  | **< 0.001** | **< 0.001** |  | **< 0.001** | **< 0.001** |

Abbreviations: CI, confidence interval; eGFR, estimated glomerular filtration rate; MAFLD, metabolic associated fatty liver disease; OR, odds ratio; T2DM, type 2 diabetes mellitus; UACR, urinary albumin to creatinine ratio. ^†^Age, sex and BMI adjusted. ^‡^Adjusted for age, sex, BMI, education level, occupation, alcohol drinking status, tea drinking status, HBV infection and MET/day. ^§^Absence of MAFLD. ^¶^Absence of T2DM. ^††^Presence of MAFLD. ^‡‡^Presence of T2DM. ^§§^Newly diagnosed T2DM is defined by laboratory indicators without pre-existing T2DM, meaning relative short duration of T2DM. ^¶¶^Pre-existing T2DM is defined as self-reported T2DM and use of antidiabetic drug, meaning relative long duration of T2DM. Boldface type indicates statistical significance (P value < 0.05).

Supplementary table 5. Association of MAFLD and metabolic goal achievement with eGFR and UACR

|  | No. | eGFR | |  | UACR | |
| --- | --- | --- | --- | --- | --- | --- |
|  |  | Model 2^†^  (β and 95% CI) | Model 3^‡^  (β and 95% CI) |  | Model 2^†^  (β and 95% CI) | Model 3^‡^  (β and 95% CI) |
| Non-MAFLD | 3716 | ref | ref |  | ref | ref |
| MAFLD with HbA1c<6.5% | 1330 | **0.95 (0.27,1.63)** | **1.02 (0.34,1.71)** |  | **0.10 (0.03,0.16)** | **0.10 (0.04,0.16)** |
| MAFLD with HbA1c≥6.5% | 548 | **3.35 (2.42,4.29)** | **2.80 (1.67,3.93)** |  | **0.61 (0.52,0.70)** | **0.34 (0.23,0.45)** |
| Non-MAFLD | 3716 | ref | ref |  | ref | ref |
| MAFLD with BP<140/90 mmHg | 794 | **1.47 (0.68,2.27)** | **1.34 (0.54,2.14)** |  | -0.05 (-0.13,0.02) | -0.12(-0.19,-0.04) |
| MAFLD with BP≥140/90 mmHg | 1084 | **1.73 (0.99,2.48)** | **1.40 (0.63,2.16)** |  | **0.49 (0.42,0.56)** | **0.38 (0.31,0.45)** |
| Non-MAFLD | 3716 | ref | ref |  | ref | ref |
| MAFLD with LDL<100 mg/dL | 279 | **2.02 (0.81,3.24)** | **1.69 (0.46,2.91)** |  | **0.34 (0.22,0.45)** | **0.22 (0.10,0.33)** |
| MAFLD with LDL≥100 mg/dL | 1599 | **1.55 (0.90,2.20)** | **1.32 (0.66,1.99)** |  | **0.22 (0.16,0.29)** | **0.14 (0.07,0.20)** |
|  |  |  |  |  |  |  |
| Non-MAFLD | 3716 | ref | ref |  | ref | ref |
| MAFLD |  |  |  |  |  |  |
| HbA1c≥6.5%&BP≥140/90 mmHg | 380 | **3.07 (1.99,4.15)** | **2.53 (1.28,3.78)** |  | **0.80 (0.70,0.90)** | **0.53 (0.42,0.65)** |
| HbA1c<6.5%&BP≥140/90 mmHg | 704 | **1.09 (0.24,1.93)** | **1.12 (0.27,1.97)** |  | **0.33 (0.25,0.41)** | **0.33 (0.25,0.4)** |
| HbA1c≥6.5%&BP<140/90 mmHg | 168 | **4.00 (2.49,5.52)** | **3.44 (1.79,5.08)** |  | **0.25 (0.11,0.39)** | -0.02 (-0.17,0.13) |
| HbA1c<6.5%&BP<140/90 mmHg | 626 | 0.81 (-0.05,1.67) | **0.92 (0.06,1.78)** |  | **-0.14 (-0.22,-0.05)** | **-0.13(-0.21,-0.05)** |
|  |  |  |  |  |  |  |
| Non-MAFLD | 3716 | ref | ref |  | ref | ref |
| MAFLD |  |  |  |  |  |  |
| 0 achieved metabolic goal^§^ | 323 | **3.06 (1.91,4.22)** | **2.16 (0.88,3.45)** |  | **0.75 (0.64,0.85)** | **0.49 (0.37,0.61)** |
| 1 achieved metabolic goals^§^ | 800 | **1.72 (0.91,2.53)** | **1.54 (0.72,2.37)** |  | **0.34 (0.26,0.41)** | **0.28 (0.20,0.35)** |
| 2-3 achieved metabolic goals^§^ | 755 | **0.97 (0.16,1.78)** | **1.02 (0.22,1.83)** |  | -0.04 (-0.12,0.03) | -0.05 (-0.13,0.02) |

Abbreviations: BP, blood pressure; CI, confidence interval; eGFR, estimated glomerular filtration rate; HbA1c, glycated hemoglobin A1c; MAFLD, metabolic associated fatty liver disease; T2DM, type 2 diabetes mellitus; UACR, urinary albumin to creatinine ratio. ^†^Age, sex and BMI adjusted. ^‡^Adjusted for age, sex, BMI, education level, occupation, alcohol drinking status, tea drinking status, HBV infection, T2DM and MET/day. ^§^Achieved metabolic goal is defined as HbA1c<6.5%, SBP/DBP<140/90 mmHg and LDL-C<100 mg/dL. Boldface type indicates statistical significance (P value < 0.05).

Supplementary table 6. Association of MAFLD and metabolic goal achievement with albuminuria

|  | No. | albuminuria | Model 1^†^  (OR and 95% CI) | Model 2^‡^  (OR and 95% CI) | Model 3^§^  (OR and 95% CI) |
| --- | --- | --- | --- | --- | --- |
| Non-MAFLD | 3716 | 261 | 1.00 (ref) | 1.00 (ref) | 1.00 (ref) |
| MAFLD with HbA1c<6.5% | 1330 | 140 | **1.56 (1.26,1.93)** | 1.25 (0.98,1.59) | 1.28 (1.00,1.63) |
| MAFLD with HbA1c≥6.5% | 548 | 160 | **5.46 (4.37,6.82)** | **3.67 (2.84,4.73)** | **1.89 (1.39,2.56)** |
| Non-MAFLD | 3716 | 261 | 1.00 (ref) | 1.00 (ref) | 1.00 (ref) |
| MAFLD with BP<140/90 mmHg | 794 | 59 | 1.06 (0.79,1.43) | 0.91 (0.67,1.24) | 0.74 (0.54,1.02) |
| MAFLD with BP≥140/90 mmHg | 1084 | 241 | **3.78 (3.13,4.58)** | **2.69 (2.14,3.36)** | **2.05 (1.62,2.59)** |
| Non-MAFLD | 3716 | 261 | 1.00 (ref) | 1.00 (ref) | 1.00 (ref) |
| MAFLD with LDL<100 mg/dL | 279 | 50 | **2.89 (2.08,4.02)** | **2.22 (1.56,3.17)** | **1.59 (1.11,2.30)** |
| MAFLD with LDL≥100 mg/dL | 1599 | 250 | **2.45 (2.04,2.95)** | **1.81 (1.46,2.24)** | **1.42 (1.14,1.78)** |
|  |  |  |  |  |  |
| Non-MAFLD | 3716 | 261 | 1.00 (ref) | 1.00 (ref) | 1.00 (ref) |
| MAFLD |  |  |  |  |  |
| HbA1c≥6.5%&BP≥140/90 mmHg | 380 | 132 | **7.05 (5.51,9.01)** | **4.78 (3.62,6.31)** | **2.48 (1.80,3.43)** |
| HbA1c<6.5%&BP≥140/90 mmHg | 704 | 109 | **2.43 (1.91,3.08)** | **1.83 (1.40,2.40)** | **1.87 (1.43,2.45)** |
| HbA1c≥6.5%&BP<140/90 mmHg | 168 | 28 | **2.65 (1.73,4.05)** | **1.96 (1.26,3.06)** | 1.00 (0.62,1.61) |
| HbA1c<6.5%&BP<140/90 mmHg | 626 | 31 | 0.69 (0.47,1.01) | **0.62 (0.42,0.92)** | **0.64 (0.43,0.95)** |
| Non-MAFLD | 3716 | 261 | 1.00 (ref) | 1.00 (ref) | 1.00 (ref) |
| MAFLD |  |  |  |  |  |
| 0 achieved metabolic goal^¶^ | 323 | 108 | **6.65 (5.11,8.65)** | **4.39 (3.28,5.88)** | **2.33 (1.67,3.23)** |
| 1 achieved metabolic goals^¶^ | 800 | 137 | **2.74 (2.19,3.42)** | **2.01 (1.56,2.58)** | **1.71 (1.32,2.21)** |
| 2-3 achieved metabolic goals^¶^ | 755 | 55 | 1.04 (0.77,1.41) | 0.90 (0.65,1.24) | 0.87 (0.63,1.20) |

Abbreviations: BP, blood pressure; CI, confidence interval; HbA1c, glycated hemoglobin A1c; MAFLD, metabolic associated fatty liver disease; OR, odds ratio. ^†^Unadjusted model. ^‡^Age, sex and BMI adjusted. ^§^Adjusted for age, sex, BMI, education level, occupation, alcohol drinking status, tea drinking status, HBV infection, T2DM and MET/day. ^¶^Achieved metabolic goal is defined as HbA1c<6.5%, SBP/DBP<140/90 mmHg and LDL-C<100 mg/dL. Boldface type indicates statistical significance (P value < 0.05).
